# GABAergic responses of mammalian ependymal cells in the central canal neurogenic niche of the postnatal spinal cord^☆^

**DOI:** 10.1016/j.neulet.2013.07.007

**Published:** 2013-10-11

**Authors:** Laura F. Corns, Jim Deuchars, Susan A. Deuchars

**Affiliations:** School of Biomedical Sciences, University of Leeds, Leeds, United Kingdom

**Keywords:** Spinal cord, Ependymal cell, Gamma amino butyric acid, Gap junction, Electrophysiology

## Abstract

The area surrounding the central canal of the postnatal mammalian spinal cord is a highly plastic region that exhibits many similarities to other postnatal neurogenic niches, such as the subventricular zone. Within this region, ependymal cells have been identified as neural stem cells however very little is known about their properties and how the local environment, including neurotransmitters, is capable of affecting them. The neurotransmitter GABA is present around the central canal and is known to affect cells within other postnatal neurogenic niches. This study used whole cell patch clamp electrophysiology and intracellular dye-loading in in vitro Wistar rat spinal cord slices to characterise ependymal cells and their ability to respond to GABA. Ependymal cells were defined by their passive response properties and low input resistances. Extensive dye-coupling was observed between ependymal cells; this was confirmed as gap junction coupling using the gap junction blocker, 18β-glycyrrhetinic acid, which significantly increased the input resistance of ependymal cells. GABA depolarised all ependymal cells tested; the partial antagonism of this response by bicuculline and gabazine indicates that GABA_A_ receptors contribute to this response. A lack of effect by baclofen suggests that GABA_B_ receptors do not contribute to the GABAergic response. The ability of ependymal cells to respond to GABA suggests that GABA could be capable of influencing the proliferation and differentiation of cells within the neurogenic niche of the postnatal spinal cord.

## Highlights

• Extensive dye-coupling occurs between mammalian spinal cord ependymal cells.
• GABA depolarised all spinal cord ependymal cells tested.
• GABA effects were mediated by GABA_A_ receptors but not GABA uptake transporters.

## 1 Introduction

The functional characteristics of ependymal cells surrounding the central canal (CC) of the mammalian spinal cord are currently poorly understood. Given the growing belief that the CC region is a postnatal neurogenic niche and that ependymal cells could be acting as neural stem/progenitor cells [2,5] it is important to understand the basic characteristics of ependymal cells and how they respond to changes in their environment. A recent study has made the first steps towards characterising ependymal cells using electrophysiology in the rat, demonstrating that they have a low input resistance (124 ± 24 MΩ), a resting membrane potential of −84 ± 2 mV and passive response properties to current pulses [16]. Dye-coupling was also observed between these cells and immunohistochemistry revealed the presence of connexin 43 indicating that gap junction coupling occurs between ependymal cells [16]. Gap junction coupling is known to influence proliferation in the developing CNS and is also observed between neural progenitor cells in the postnatal Subventricular zone (SVZ) [3,9]; it could be performing a similar function here.

There are currently no studies investigating the effect of neurotransmitters on ependymal cells surrounding the CC of the mammalian spinal cord. A recent study by Reali et al. [20], explored the effects of GABA on brain lipid binding protein (BLBP) immunoreactive progenitor cells that are found surrounding the CC of the turtle spinal cord; these cells appear to be similar to the radial ependymal cells or potential radial glia that surround the mammalian CC [16,17]. GABA depolarised these progenitor cells inducing increases in intracellular Ca^2+^, suggesting that GABA is capable of stimulating long lasting intracellular effects within these cells [20]. The response to GABA was mediated by either GABA_A_ receptors, the GABA uptake transporter, GAT3, or a combination of both [20], highlighting variation within the population of progenitor cells. Neural stem and progenitor cells within other mammalian postnatal neurogenic niches such as the subventricular zone (SVZ) or the dentate gyrus (DG) also respond to GABA and as a result GABA is capable of influencing the proliferation and differentiation of these cells [12,23].

Given this conserved ability of neural stem and progenitor cells to be able to respond to GABA and the presence of GABAergic terminals forming synapses with ependymal cells surrounding the mammalian CC [14], it is highly likely that GABA is affecting these cells. As ependymal cells are an endogenous source of new cells within the postnatal mammalian spinal cord, it is pertinent to increase both our knowledge about how mammalian ependymal cells communicate between themselves and whether they can respond to external stimuli, such as GABA. This study used whole cell patch clamp electrophysiology and intracellular dye-loading to demonstrate that ependymal cells in the postnatal spinal cord were capable of responding to GABA and that this response was in part mediated by GABA_A_ receptors.

## 2 Materials and methods

### 2.1 Animals

Wistar rats (P9-21) were used in line with the Animals Scientific Act (1986) and the ethical standards set out by the University of Leeds Ethical Review Committee by individuals with Home Office approval. Every effort was made to minimise the number of animals used and their suffering.

### 2.2 Slice preparation

Animals were anaesthetised by administration of pentabarbitone (60 mg/kg) I.P and perfused transcardially with ice cold sucrose aCSF (mM): sucrose (217), NaHCO_3_ (26), KCl (3), MgSO_4_·7H_2_O (2), NaH_2_PO_4_ (2.5), glucose (10), CaCl_2_ (1), equilibrated with 95% O_2_ and 5% CO_2_. The spinal cord was removed after a dorsal laminectomy, the dura and pia mater removed and the lower thoracic spinal cord embedded in agar before cutting 300 μm thick transverse slices using a vibrating microtome (Leica VT1200s, Leica Microsystems, UK). Slices were transferred to a holding chamber containing aCSF (95% O_2_: 5% CO_2_; mM): NaCl (124), NaHCO_3_ (26), KCl (3), MgSO_4_·7H_2_O (2), NaH_2_PO_4_ (2.5), glucose (10), CaCl_2_ (2), and left to equilibrate in this solution at room temperature for 30–60 min.

### 2.3 Whole cell patch clamp electrophysiology

Recordings were made at room temperature. Viable ependymal cells were viewed at 60× magnification using differential interference contrast (DIC) imaging and QCapture Pro software via a camera (QImaging Rolera-XR, Q imaging, Scientifica, UK) attached to the microscope (Olympus BX50W1). Whole cell patch clamp recordings were made with microelectrodes filled with intracellular solution (mM): K gluconate (110), EGTA (11), MgCl_2_ (2), CaCl_2_ (0.1), HEPES (10), Na_2_ATP (2), NaGTP (0.3). Neurobiotin (0.5%; Vector Laboratories, USA) and tetramethylrodamine (0.02%; Invitrogen, USA) were added to allow visualisation of the neurone post-recording. *E*_Cl_ calculated using the Nernst equation from the stated solutions was −103 mV. Current clamp recordings (to enable characterisation of the cells) were performed using an Axopatch-1D amplifier (Molecular Devices) and signals were captured by pClamp9 software (Molecular Devices). The membrane potential measurements were not corrected for the liquid junction potential. To characterise voltage responses, depolarising and hyperpolarising current pulses of 1 s duration (ranging from −50 pA to +50 pA) were applied. The input resistance was determined by the size of the hyperpolarisation in response to the injection of −50 pA current pulses.

### 2.4 Drugs

All drugs were prepared as stock solutions in water unless otherwise stated and bath applied using a gravity perfusion system. GABA (200 μM; Sigma–Aldrich) was bath applied alone or co-applied with bicuculline methochloride (100 μM, Ascent Scientific, UK), gabazine (2.5 μM, dissolved in DMSO, final concentration 0.1%, Sigma–Aldrich), guvacine hydrochloride (50 μM, Tocris Bioscience, UK) or ±nipecotic acid (300–900 μM, Tocris Bioscience, UK). (R) – baclofen (10 μM, Tocris Bioscience, UK) was applied alone to determine whether cells responded to activation of GABA_B_ receptors. 18β-glycyrrhetinic acid (18β-GA) potassium salt (100 μM, Sigma–Aldrich) was bath applied to investigate the presence of gap junctions. On several occasions, GABA (500 μM) was pressure ejected using a PV800 pneumatic picopump (World Precision Instruments Ltd., UK, 10 psi; 500 ms pressure duration) from a separate micropipette placed in the vicinity of the recorded cell.

### 2.5 Analysis

Changes in membrane potential and input resistance were recorded before, during and after drug applications and all data expressed as mean ± standard error (S.E.). Drug effects were determined using paired *t*-tests or one-way ANOVAs with post hoc Bonferroni tests and differences between drug effects determined using independent *t*-tests.

### 2.6 Morphological identification of ependymal cells

Slices were imaged at the end of recordings using epifluorescence with a filter of 555 nm. The recording electrode was removed and the slice fixed in 4% PFA + 0.25% glutaraldehyde for detailed post hoc visualisation. Slices were washed in 0.1 M phosphate buffer, gelatine embedded and resectioned into 50 μm sections using a vibrating microtome. Sections were permeabilised in phosphate buffered saline (PBS) containing 0.1% triton X-100 before being incubated in extraavidin peroxidase (1:250 in PBS) for 36–72 h then washed in 0.1 M PBS. Cells were visualised by incubating in diaminobenzidine (DAB; 0.5 mg/ml in tris buffer + 1% H_2_O_2_) until the staining was visible.

## 3 Results

### 3.1 Ependymal cells in the mammalian spinal cord have passive response properties and are coupled by gap junctions

**Fig. 1:**
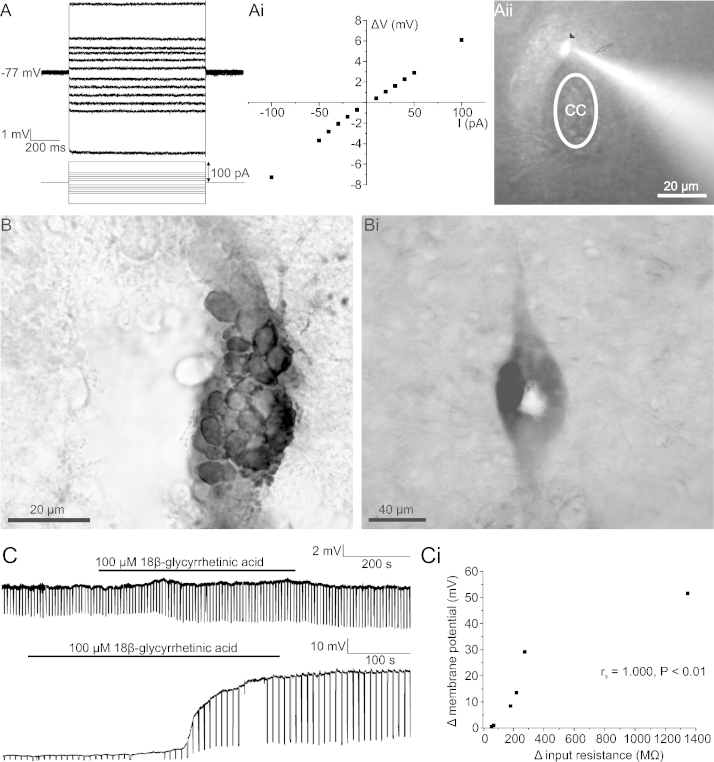
Basic electrophysiological characteristics of ependymal cells. (A) Current clamp recording of an ependymal cell responding to injection of positive and negative current pulses. The change in voltage (Δ*V*) with respect to the current injected is plotted for the same ependymal cell (i). The recorded cell was confirmed as an ependymal cell post-recording by visualisation of rhodamine, indicated by the arrowhead; the arrow indicates the rhodamine-filled patch electrode (ii). (B) Visualisation of Neurobiotin by DAB revealed the extent of dye-coupling between ependymal cells. (C) Bath application of 18β-GA (100 μM) in two separate ependymal cells, downward deflections are voltage responses to −50 pA current pulses. Correlation between the change in input resistance and the change in membrane potential in response to the bath application of 18β-glycyrrhetinic acid, *r*_s_ = Spearman's Rank Order Correlation Co-efficient (i).

The ependymal cells recorded had a resting membrane potential of −76 ± 0.5 mV (*n* = 79), small input resistances (96 ± 13 MΩ; *n* = 79) and displayed no spontaneous or evoked activity (Fig. 1A). There was a linear relationship between the change in voltage in response to a current pulse and the size of the current pulse (Fig. 1Ai); this demonstrates that ependymal cells have passive responses indicative of a lack of voltage-gated ion channels. A Spearman's rank order correlation test found a significant but weak positive correlation between the resting membrane potential and the input resistance (*r*_s_ (79) = 0.337, *P* = 0.002), demonstrating that ependymal cells with more negative resting membrane potentials generally have smaller input resistances. Cells were confirmed as ependymal cells by a lack of processes when visualised by rhodamine or Neurobiotin post-recording (*n* = 79; Fig. 1Aii).

Neurobiotin, which was present within the intracellular solution, is gap junction permeable and allows coupling between cells to be observed [24]. The visualisation of Neurobiotin with DAB revealed that the ependymal cells were highly coupled (*n* = 28 out of 28 recovered for Neurobiotin; Fig. 1B). There was variation in the size of the dye-coupled clusters, with some clusters covering almost the whole CC region (Fig. 1Bi). Post-recording visualisation of cells with rhodamine, which is not gap junction permeable [7], showed no coupling of cells, indicating that there was no spillage of tracers. To confirm that the transfer of dye was through gap junctions, the non-selective gap junction blocker 18β-GA (100 μM; [6]) was bath applied. 18β-GA increased the input resistance of ependymal cells by an average of 360 ± 201 MΩ (*n* = 6; Fig. 1C), with increases ranging from 57 MΩ to 1346 MΩ. A Wilcoxon Signed Rank Test determined that this increase was significant (*Z* = −2.201, *P* = 0.028). The range of changes in input resistance suggests that the ependymal cells are gap junction coupled to different degrees, supporting the dye-coupled data.18β-GA also depolarised ependymal cells to different degrees (0–52 mV). A Spearman's Rank Order correlation found a strong, significant correlation between the change in input resistance and the change in membrane potential (*r*_s_ (4) = 1.000, *P* < 0.01; Fig. 1Ci). This suggests that the depolarisation that occurs in response to the application of 18β-GA is related to blockade of gap junctions and not a non-gap junction related effect of 18β-GA.

### 3.2 Ependymal cells of the mammalian spinal cord respond to GABA

**Fig. 2:**
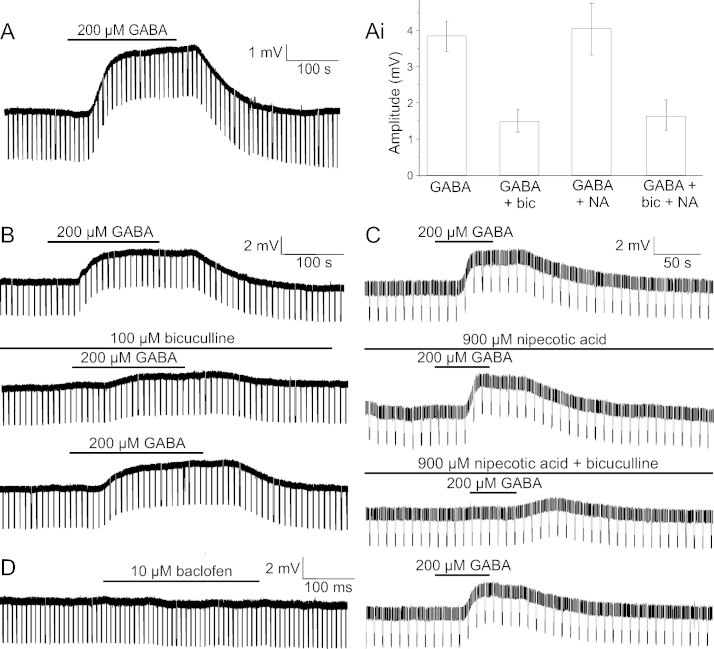
Ependymal cells are depolarised by GABA. (A) Current clamp recording during bath application of GABA (200 μM) to an ependymal cell. Group data for responses to GABA alone or in the presence of bicuculline (bic) and/or nipecotic acid (NA) (i). (B) Current clamp recordings demonstrating that the depolarisation in response to GABA (200 μM) is partially antagonised by bicuculline (100 μM) in a reversible manner. (C) The depolarisation in response to GABA (200 μM) is not affected by nipecotic acid (900 μM), although a reversible partial antagonism is seen in this cell with nipecotic acid and bicuculline (100 μM). (D) Application of baclofen (10 μM). The downward voltage deflections are in response to the regular injection of −50 pA current pulses.

Bath application of GABA (200 μM) resulted in a significant, reversible depolarisation in ependymal cells (3.85 ± 0.37 mV; *n* = 18; *P* < 0.001; Fig. 2A), with responses ranging from 2.03 mV to 6.46 mV. Although there was a trend towards a decrease in input resistance on application of GABA (71.10 ± 12.17 MΩ to 68.81 ± 10.75 MΩ; *n* = 10), the decrease was not significant (*P* = 0.173; data not shown). Focal pressure ejection of GABA (500 μM) depolarised ependymal cells (4.40 ± 2.68 mV; *n* = 3), with a time to onset of 140 ± 28 ms (*n* = 3) and a time from onset to peak response of 848 ± 62 ms (*n* = 3; data not shown).

To determine which receptors were mediating GABAergic responses in ependymal cells, GABA (200 μM) was bath applied in the presence of GABA_A_ antagonists, bicuculline (100 μM) or gabazine (2.5 μM). There was no significant difference in the input resistance of ependymal cells (78.68 ± 14.08 MΩ to 79.94 ± 14.99 MΩ; *n* = 4) during co-application of bicuculline and GABA (*F* (2, 6) = 0.310, *P* = 0.745). Similar to application of GABA alone, the application of GABA in the presence of bicuculline significantly depolarised ependymal cells (1.45 ± 0.27 mV; *n* = 7; Fig. 2Ai and B). However, the size of this depolarisation was significantly smaller than that in GABA alone (*t* (3) = 3.685, *P* = 0.035), with GABAergic responses in bicuculline being 38 ± 10% (*n* = 7) of the initial GABA response. Bath application of gabazine (2.5 μM) also antagonised GABA responses, with a 37 ± 11% (*n* = 2) decrease in the size of the depolarisation. The effects of both bicuculline and gabazine were reversible (Fig. 2B).

It was investigated whether the remainder of the GABAergic response could be mediated by GABA transporters, as observed in ependymal cells of turtle spinal cord [20]. The bath application of GABA in the presence of nipecotic acid (900 μM) or guvacine (50 μM), non-selective GABA transporter blockers, resulted in a significant depolarisation of ependymal cells (4.11 ± 0.67 mV; *n* = 7; Fig. 2C). The size of this depolarisation was not significantly different (*P* = 0.34) from that of ependymal cells responding to GABA under control conditions (4.17 ± 0.67 mV; *n* = 7; Fig. 2 Ai and C). Furthermore, co-application of either guvacine or nipecotic acid and bicuculline still failed to completely block the response mediated by bath application of GABA (1.55 ± 0.63 mV; *n* = 3; Fig. 2Ai and C).

As the GABA_A_ antagonists could not entirely antagonise the GABAergic response, the effect of baclofen was investigated. There was, however, no significant change in the membrane potential (−78.38 ± 1.45 mV to −78.57 ± 1.59 mV; *n* = 3) during the bath application of baclofen (*F* (2, 4) = 2.601, *P* = 0.189; Fig. 2D). Neither was there a significant change in the input resistance (68.90 ± 13.38 MΩ to 70.55 ± 13.92 MΩ; *n* = 3) during the application of baclofen (*F* (2, 4) = 1.449, *P* = 0.366; Fig. 2D).

## 4 Discussion

This study provides an electrophysiological characterisation of ependymal cells surrounding the CC and is the first study to demonstrate that ependymal cells in this area within the postnatal mammalian spinal cord respond to GABA. Ependymal cells displayed typical characteristics of glial cells, with no spontaneous or evoked activity, indicating a lack of voltage-gated channels. Dye coupling with Neurobiotin following intracellular loading confirmed reports that ependymal cells are coupled and the gap junction blocker 18β-glycrrhetinic acid established that this coupling was mediated by gap junctions. Ependymal cells consistently depolarised to GABA, an effect partially antagonised by GABA_A_ receptor antagonists, bicuculline and gabazine, but the remainder of the response was not decreased by GABA transporter blockers, nor was the response mimicked by the GABA_B_ agonist baclofen. The ability of these cells, which are considered to be neural stem cells, to respond to GABA is extremely pertinent and highlights the need for further studies investigating how GABA affects the proliferation and differentiation of these cells.

The input resistance of 96 MΩ in ependymal cells is slightly lower than that previously determined for ependymal cells in the rat spinal cord, 124 MΩ [16]. As connexin expression is known to increase steadily from P0 to adulthood in other CNS areas [10], the lower input resistance here may be due to the older animals used (P11–P21) compared to that of Marichal et al. ([16] P0–P5). The lack of spontaneous or evoked activity and the linear voltage–current relationship agrees with previous studies of rat and turtle spinal cord ependymal cells [15,16,21] and suggests that ependymal cells lack voltage-gated ion channels.

### 4.1 The relevance of gap junction coupling

This study confirmed previous reports that gap junction coupling occurs between ependymal cells of the rat spinal cord [16]. As 18β-GA is a non-selective gap junction blocker, the specific identity of the connexin subunits forming the gap junctions was not identified, however, immunohistochemistry implies that either connexin 43 [16,19] and/or connexin 45 [4] form the gap junctions between ependymal cells. The strong correlation between the change in input resistance and the change in membrane potential in response to 18β-GA indicates that the depolarisation is a direct effect of gap junction blockade rather than a non-gap junction specific effect of 18β-GA. This effect is similar to that observed in progenitor cells surrounding the turtle CC [20]. A possible reason for ependymal cells to form gap junctions is to allow the control of cellular proliferation, as seen in the embryonic neocortex and in the adult SVZ [3,11]

### 4.2 Could GABA influence ependymal cells?

The depolarisation of ependymal cells observed following bath or focal application of GABA resembles that observed in progenitor cells surrounding the CC of the turtle spinal cord [20] and in the postnatal neurogenic niches of the brain [12,23]. Given that E_GABA_ is predominantly influenced by $E_{{\text{Cl}}^{-}}$, which was −103 mV in this study, a hyperpolarisation rather than a depolarisation would have been expected. Although the presence of the Na^+^–K^+^–2Cl^−^ co-transporter (NKCC1) in ependymal cells would not generally be enough to overcome the low intracellular Cl^−^ concentration imposed by the intracellular solution within the patch pipette, if the NKCC1 channels were expressed in close proximity to GABA_A_ receptors in the cell membrane, a local accumulation of intracellular Cl^−^ could explain the depolarisation. The high degree of gap junction coupling could also allow the movement of Cl^−^ into the recorded cell, however, this is unlikely to be sufficient to raise intracellular Cl^−^ concentration. Most likely, the depolarisation resulted from an intense activation of GABA receptors, as commonly observed [18,22]. This prolonged activation of GABA_A_ receptors can lead to an imbalance of HCO^3−^ efflux and Cl^−^ influx that shifts E_GABA_ away from $E_{{\text{Cl}}^{-}}$ towards $E_{{\text{HCO}}^{\text{3-}}}$, which was −12 mV here. A final speculation was that the depolarisation could in part be a result of a contribution by GABA uptake transporters which transport GABA back into the cells with two Na^+^ ions and one Cl^−^ ion, thus carrying a net positive charge into the cell [13]. The lack of effect of nipecotic acid or guvacine, non-selective GABA uptake transporter blockers, in this study suggests that this is not the case. It also highlights a difference between the ependymal cells of the turtle spinal cord, where GABA transporters contributed to the GABAergic response and those investigated here in the rat spinal cord. Other similarities were observed between the two species, including the presence of GABA_A_ receptors mediating the GABAergic response. However, in rats it remains to be determined what is mediating the bicuculline resistant component of the GABAergic response.

The endogenous source of GABA is likely to be the GABAergic terminals that synapse with ependymal cells [14]. These terminals could originate from either local GABAergic interneurons or from neighbouring GABAergic cerebrospinal fluid contacting neurones (CSFcNs) that are in the subependymal layer of the CC [1]. The CSFcNs may release GABA into the CSF from their CSF-contacting processes, enabling widespread distribution of GABA to ependymal cells.

The fact that ependymal cells have properties of neural stem cells [2,8,25] and GABA influences the proliferation and differentiation of neural stem cells in other neurogenic niches [12,23] suggests that GABA could be influencing the proliferation and/or the differentiation of ependymal cells surrounding the CC. In the postnatal neurogenic niches of the brain, GABA appears to reduce the proliferation of neural stem/progenitor cells and induce differentiation to produce more newborn neurones [12,23]. Unlike the postnatal neurogenic niches of the mammalian brain and lower vertebrate spinal cord, ependymal cells undergo only symmetrical division to maintain the ependymal cell population under physiological conditions [8]. If cells within the CC area respond to GABA in a similar way to the SVZ and DG, the proliferation of ependymal cells rather than differentiation suggests a lack of endogenous GABA under physiological conditions. It is possible that following an injury or the onset of a pathological condition, GABA could be released around this area, limiting proliferation and promoting differentiation. If this is the case, being able to manipulate this GABAergic modulation would enable a greater control over the neurogenic capacity of this area.

## 5 Conclusion

This study demonstrates that ependymal cells surrounding the CC of the postnatal mammalian spinal cord are capable of responding to the neurotransmitter GABA. Ependymal cells could be more integrated into the spinal cord circuitry than previously expected and may be capable of responding to changes in the environment, with potential consequences for the neurogenic capacity of the area.
